# Corrigendum: Oroxylin a Inhibits the Protection of Bone Marrow Microenvironment on CML Cells Through CXCL12/CXCR4/P-gp Signaling Pathway

**DOI:** 10.3389/fonc.2019.00990

**Published:** 2019-09-27

**Authors:** Hanbo Cao, Wenjun Li, Yizhou Zhou, Renxiang Tan, Yue Yang, You Zhou, Qinglong Guo, Li Zhao

**Affiliations:** ^1^State Key Laboratory of Natural Medicines, Jiangsu Key Laboratory of Carcinogenesis and Intervention, China Pharmaceutical University, Nanjing, China; ^2^State Key Laboratory Cultivation Base for TCM Quality and Efficacy, Nanjing University of Chinese Medicine, Nanjing, China

**Keywords:** bone marrow environment, CXCL12/CXCR4, oroxylin A, Imatinib (IM), β-catenin/P-gp

In the original article, there was a mistake in Figure 3H as published. Due to carelessness, the picture of the “k562-indo group” was accidentally placed in the “ku812-indo group”. The corrected Figure 3H appears below.

**Figure 3:**
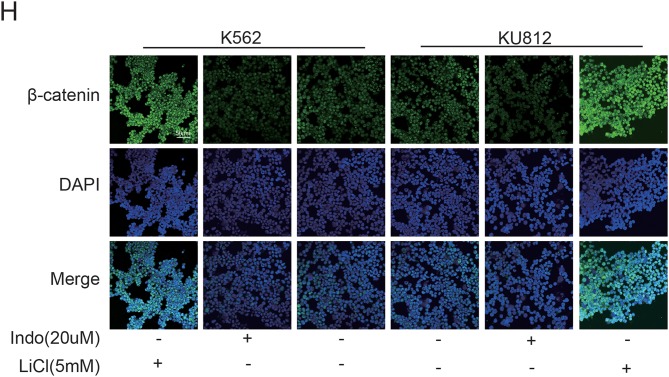


The authors apologize for this error and state that this does not change the scientific conclusions of the article in any way. The original article has been updated.

